# Low apolipoprotein A1 was associated with increased risk of cancer mortality in patients following percutaneous coronary intervention: A 10‐year follow‐up study

**DOI:** 10.1002/ijc.34164

**Published:** 2022-07-07

**Authors:** Hiroki Nishiyama, Takehiro Funamizu, Hiroshi Iwata, Hirohisa Endo, Yuichi Chikata, Shinichiro Doi, Hideki Wada, Ryo Naito, Manabu Ogita, Yoshiteru Kato, Iwao Okai, Tomotaka Dohi, Takatoshi Kasai, Kikuo Isoda, Shinya Okazaki, Katsumi Miyauchi, Tohru Minamino

**Affiliations:** ^1^ Department of Cardiovascular Biology and Medicine Juntendo University Graduate School of Medicine Tokyo Japan; ^2^ Department of Cardiology Juntendo University Shizuoka Hospital Shizuoka Japan; ^3^ Department of Cardiology Juntendo University Nerima Hospital Tokyo Japan

**Keywords:** apolipoprotein A1, cancer mortality, high‐density lipoprotein cholesterol, percutaneous coronary intervention

## Abstract

Previous studies showed that elevated apolipoprotein A1 (ApoA1) and high‐density lipoprotein cholesterol (HDL‐C) predicted reduced risk of cardiovascular‐related (CV) mortality in patients following percutaneous coronary intervention (PCI). Nevertheless, as the association between ApoA1 and cancer mortality in this population has been rarely addressed, our study aimed to evaluate prognostic impact of ApoA1 on multiple types of cancer mortality after PCI. This is a retrospective analysis of a single‐center prospective registry database of patients who underwent PCI between 2000 and 2018. The present study enrolled 3835 patients whose data of serum ApoA1 were available and they were divided into three groups according to the tertiles of the preprocedural level of ApoA1. The outcome measures were total, gastrointestinal, and lung cancer mortalities. The median and range of the follow‐up period between the index PCI and latest follow‐up were 5.9 and 0‐17.8 years, respectively. Consequently, Kaplan‐Meier analyses showed significantly higher rates of the cumulative incidences of total, gastrointestinal, and lung cancer mortality in the lowest ApoA1 tertile group compared to those in the highest. In contrast, there were no significant differences in all types of cancer mortality rates in the groups divided by the tertiles of HDL‐C. Multivariable Cox proportional hazard regression analysis adjusted by cancer‐related prognostic factors, such as smoking status, identified the elevated ApoA1 as an independent predictor of decreased risk of total and gastrointestinal cancer mortalities. Our study demonstrates the prognostic implication of preprocedural ApoA1 for predicting future risk of cancer mortality in patients undergoing PCI.

## Graphical abstract

What's new?

Cancer mortality has become one of the major clinical concerns in patients undergoing percutaneous coronary intervention (PCI). Apolipoprotein A plays a central role in the antiatherosclerotic effects of high‐density lipoprotein cholesterol and is also associated with reduced incidence of cancer and cancer mortality in the general population. The present study demonstrates that reduced preprocedural apolipoprotein A1 level is independently associated with increased risk of total and gastrointestinal cancer mortality following PCI. The findings indicate that serum apolipoprotein A1 level could be a useful prognostic indicator of cancer mortality in patients undergoing PCI.

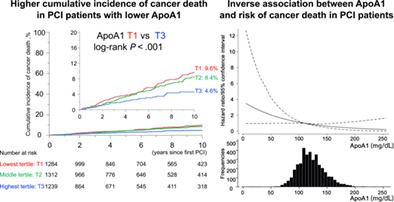

**Abbreviations**
ApoA1: apolipoprotein A1
CAD: coronary artery disease
GI: gastrointestinal
HDL‐C: high density lipoprotein cholesterol
ICD‐10: International Statistical Classification of Diseases and Related Health Problems, Tenth Revision
LDL‐C: low density lipoprotein cholesterol
MACE: major adverse cardiovascular events
PCI: percutaneous coronary intervention

## 1 INTRODUCTION

Percutaneous coronary intervention (PCI) has become the most common coronary revascularization strategy, as its clinical indication has expanded due to marked procedural and technological advancements for 40 years since its introduction.^1^ In accordance with the increasing number of patients who undergo PCI, long‐term outcomes following PCI have gathered intense clinical interest. Most of the interventional and observational studies in patients with coronary artery disease (CAD) have involved the composite of “major adverse cardiovascular events (MACE),” which were generally composed of cardiovascular‐related mortality, nonfatal myocardial infarction, and nonfatal stroke.^2^, ^3^ However, the proportion of cardiovascular‐related mortality to all‐cause death has been falling as a consequence of substantial advancements in evidence‐based preventative strategies to control atherosclerotic risk factors,^4^ while there has been no significant decline in the number of cardiovascular deaths after PCI due to expansion of the PCI indication for patients at higher‐risk.^5^, ^6^, ^7^ Recent evidence has indicated a sharp rise of long‐term noncardiovascular‐related deaths after PCI, and cancer‐related mortality in particular.^8^, ^9^ Therefore, in light of the long‐term outcomes of patients following PCI, the morbidity and mortality of cancer have become major clinical concerns.^10^, ^11^ At the same time, numerous evidence has indicated that patients with a cancer history at the time of PCI have higher risks of subsequent all‐cause and cardiovascular death.^12^, ^13^

High density lipoprotein (HDL) has been ascribed to have diverse atheroprotective properties including antioxidant and antiinflammatory functions^14^ and low serum HDL cholesterol (HDL‐C) has been a long‐standing established risk factor for coronary artery disease.^15^ Moreover, a major constituent protein of HDL‐C, apolipoprotein A (ApoA1 and ApoA2), has been considered to play a central role in the antiatherosclerotic effects of HDL.^16^ In addition to atherosclerotic perspectives, the mechanistic insights of cholesterol metabolism on carcinogenesis have also been a topic of interest in experimental investigations.^17^, ^18^, ^19^ Moreover, cohort studies in particular have indicated that levels of HDL and ApoA1 were inversely associated with the incidence of cancer and cancer mortality in general populations.^20^, ^21^ Nevertheless, in patients with established atherosclerotic coronary artery disease who are more prone to adverse cardiovascular events, the impact of HDL and ApoA1 on cancer‐related prognosis has been rarely addressed. Therefore, our study using a single‐center prospective PCI registry database aimed to clarify the possible associations of serum levels of HDL‐C and ApoA1 with subsequent cancer mortality in this population.

## 2 MATERIALS AND METHODS

### 2.1 Participants and follow‐up duration

#### 2.1.1 Participants

Our study is a retrospective analysis of a portion of a lengthy prospective single‐center registry database of patients who underwent PCI at Juntendo University Hospital. This database was launched in February 1984 and continues until the present as of November 2021 (Juntendo Physicians' Alliance for Clinical Trial, J‐PACT). This database has prospectively recorded data regarding patient demographics, characteristics of coronary artery lesions, PCI procedures, and the devices used. Patients who underwent any PCI procedure, such as thrombectomy, balloon angioplasty, and/or the deployment of any type of coronary stent, were enrolled in the registry. Our study firstly identified patients who underwent PCI for the first time between 2000 and 2018 (n = 4885). After excluding patients whose preprocedural ApoA1 values were not available, 3835 patients were enrolled in the present study. We divided the study participants into three groups according to the tertile of the ApoA1 value, the lowest tertile (T1) group: ApoA1 ≤ 110 mg/dL (n = 1284), the middle tertile group (110 < ApoA1 ≤ 130, n = 1312), and the highest tertile group (ApoA1 > 130, n = 1239). A consort diagram of the present study is shown as Figure S1.

#### 2.1.2 Outcome measures

In this registry, the prognostic follow‐up of patients was based on chart review in patients followed by the outpatient clinic of our institution. In those who were followed by external institutions, a prognosis survey questionnaire was mailed out or conducted by phone every 5 years. In cases of no response to the survey in such patients, follow‐up was terminated at the latest time point, at which their survival at our institution was confirmed, such as the last visit date to an outpatient clinic or the last day of any hospitalization. The median and range of the follow‐up period between the index PCI and latest follow‐up were 5.9 and 0‐17.8 years, respectively. The primary outcome measure was total cancer mortality. Cancer mortality was defined as the primary cause of death due to cancer, such as death due to underlying malignant disease itself, or complications of treatment for cancer.^22^ Moreover, among total cancer mortalities, gastrointestinal (GI) cancer mortality refers to death primarily by cancers of the gastrointestinal tract (GI tract) and accessory organs of digestion, including the esophagus, stomach, liver, biliary system, pancreas, small intestine, large intestine, rectum and anus according to the International Statistical Classification of Diseases and Related Health Problems, Tenth Revision (ICD‐10) codes. The secondary endpoints were all‐cause, cardiovascular, and noncardiovascular mortalities.

### 2.2 Statistical analysis

Continuous variables are presented as the mean ± SD or median with interquartile range (IQR) in accordance with the results of the Shapiro‐Wilk normality test. Categorical variables are presented as the actual number and frequencies (%). Quantitative data across groups were compared using the ANOVA test or the Kruskal‐Wallis test. Categorical variables were compared using the Fisher‐exact test with the chi‐squared test. Unadjusted Kaplan‐Meier analysis evaluated the time to the cumulative incidences of total, GI, and lung cancer mortalities followed by the log‐rank comparisons. The prognostic implication of ApoA1 for total‐, GI‐ and lung‐cancer mortalities was evaluated by using univariate‐unadjusted and multivariable‐adjusted Cox proportional hazard regression analysis. In multivariate analyses, the following two models calculated the hazard ratios (HR) with 95% confidence intervals (CIs) for future total cancer death after PCI: Model 1 included age, sex, and any smoking history, and Model 2 additionally included conventional risk factors (hypertension, diabetes, serum low density lipoprotein cholesterol [LDL‐C] level and chronic kidney disease [estimated glomerular filtration rate (eGFR) < 60 mL/min/1.73 m^2^]) for atherosclerotic cardiovascular disease, and factors previously demonstrated or indicated to increase or decrease the risk of cancer incidence and mortality, such as body mass index (BMI),^23^, ^24^ use of angiotensin converting enzyme inhibitors/angiotensin receptor blockers (ACEI/ARB)^25^ and statins,^26^ and serum high‐sensitivity C‐reactive protein (hs‐CRP) level.^27^, ^28^ Cox proportional hazard regression analysis was employed to assess the influence of ApoA1 for each cancer mortality, since the number of lung cancer cases was limited. A cubic spline term of ApoA1 was fitted in the Cox model (Model 2) to assess how the spectrum of ApoA1 related to the HRs of total and GI cancer mortalities. The reference of serum ApoA1 level was determined as 110 mg/dL, which was the lowest tertile. A *P*‐value (*P*) <.05 was considered to indicate statistical significance. Statistical analyses were performed using JMP version 12.2 (SAS Institute, Cary, NC).

## 3 RESULTS

### 3.1 Background demographics and procedural characteristics by ApoA1 tertiles

The present study included 3835 PCI patients. The mean age was 66.4 ± 10.5 years and 82.2% were male. The background demographics and lipid parameters in accordance with the tertile of the ApoA1 level (110 and 130 mg/dL, respectively) are listed and compared in Table 1. Patients in the lowest tertile (T1) group were younger, there were more males than females, and they had a higher BMI and lower eGFR compared to those in higher tertiles (T2 and T3) of ApoA1. Those in the T1 group were more likely to have diabetes, to present with acute coronary syndrome (ACS), to have any, former and current smoking habit, and not to take statins. For lipid parameters, patients in T1 group had lower levels of HDL‐C, Apo E, and total cholesterol, and higher levels of lipoprotein(a) and LDL‐C, while there was no difference in ApoB100. Pearson correlation analysis showed a strong positive correlation between ApoA1 and HDL‐C (Pearson correlation coefficient [*r*]: .76, *P* < .001). Additionally, a marker of systematic inflammation, the serum level of hs‐CRP, was inversely correlated with serum ApoA1 level (Spearman *r* = −.27, *P* < .001).

**TABLE 1:** Baseline clinical characteristics of the study population

	Overall (n = 3835)	Lowest tertile (T1) (ApoA1 ≤ 110) (n = 1284)	Middle tertile (T2) (110 < ApoA1 ≤ 130) (n = 1312)	Highest tertile (T3) (ApoA1 > 130) (n = 1239)	*P*‐value
Age, y	66.4 ± 10.5	65.5 ± 10.9	66.5 ± 10.4	67.1 ± 10.0	**<.001**
Male, n (%)	3152 (82.2)	1150 (89.6)	1086 (82.8)	916 (73.9)	**<.001**
BMI, kg/m^2^	24.3 ± 3.5	24.6 ± 3.6	24.4 ± 3.5	23.7 ± 3.3	**<.001**
Systolic blood pressure, mm Hg	136.0 ± 23.0	133.1 ± 22.7	136.6 ± 23.0	138.3 ± 23.1	**<.001**
Hypertension, n (%)	2751 (71.7)	919 (71.6)	952 (72.6)	880 (71.0)	.68
Dyslipidemia, n (%)	2755 (71.8)	898 (69.9)	940 (71.7)	917 (74.0)	.073
Diabetes, n (%)	1681 (43.8)	623 (48.5)	587 (44.7)	471 (38.0)	**<.001**
HbA1c, %	6.3 ± 1.1	6.4 ± 1.2	6.3 ± 1.1	6.2 ± 1.0	**<.001**
History of smoking, n (%)	2487 (65.0)	910 (71.1)	852 (65.1)	725 (58.5)	**<.001**
Family history, n (%)	1081 (28.4)	363 (28.5)	390 (30.0)	328 (26.5)	.16
Presentation of ACS, n (%)	836 (21.8)	372 (29.0)	268 (20.4)	196 (15.8)	**<.001**
Number of vessels	1.8 ± 0.8	1.9 ± 0.8	1.8 ± 0.8	1.7 ± 0.8	**<.001**
RCA, n (%)	1142 (29.8)	415 (32.4)	362 (27.6)	365 (29.5)	**.028**
LAD, n (%)	1814 (47.3)	581 (45.3)	635 (48.4)	598 (48.3)	.21
LCX, n (%)	704 (18.4)	225 (17.6)	258 (19.7)	221 (17.8)	.32
LMT, n (%)	108 (2.8)	29 (2.3)	37 (2.8)	42 (3.4)	.23
SVG, n (%)	58 (1.5)	28 (2.2)	18 (1.4)	12 (1.0)	**.041**
Stent diameter, mm	3.0 ± 0.4	3.0 ± 0.4	3.0 ± 0.4	3.0 ± 0.4	**<.001**
Total stent length, mm	21 [16‐32]	20 [16‐32]	22 [16‐32]	22 [16‐32]	.99
Beta blocker, n (%)	1894 (50.1)	652 (51.6)	671 (51.8)	571 (46.7)	**.017**
CCB, n (%)	1566 (41.5)	486 (38.5)	551 (42.6)	529 (43.3)	**.031**
ACEI/ARB, n (%)	1952 (51.6)	684 (54.2)	706 (54.5)	562 (46.0)	**<.001**
Aspirin, n (%)	3520 (93.1)	1176 (93.1)	1207 (93.1)	1137 (93.0)	.99
Statins, n (%)	2388 (63.2)	714 (56.6)	838 (64.7)	836 (68.4)	**<.001**
TC, mg/dL	178.4 ± 38.7	170.5 ± 40.5	176.4 ± 36.2	188.6 ± 37.2	**<.001**
LDL‐C, mg/dL	107.3 ± 33.6	109.3 ± 36.0	106.5 ± 32.1	106.0 ± 32.5	**.028**
HDL‐C, mg/dL	44.7 ± 13.1	34.8 ± 7.3	43.3 ± 8.9	56.5 ± 12.2	**<.001**
TG, mg/dL	116.0 [86.0‐159.0]	116.5 [86.0‐160.8]	120.0 [88.0‐161.0]	113.0 [84.0‐156.0]	.064
ApoA1, mg/dL	121.9 ± 24.8	96.9 ± 12.2	120.1 ± 5.8	149.6 ± 17.4	**<.001**
ApoB100, mg/dL	89.7 ± 23.2	90.7 ± 23.9	89.8 ± 23.0	88.4 ± 22.5	**.044**
ApoE, mg/dL	4.1 ± 1.6	3.8 ± 1.9	4.0 ± 1.2	4.4 ± 1.5	**<.001**
Lipoprotein (a), mg/dL	18.3 [10.0‐33.0]	20.5 [11.0‐34.4]	17.7 [9.6‐32.0]	17.0 [8.0‐31.9]	**<.001**
Non‐FBG, mg/dL	113.5 ± 40.2	117.7 ± 46.6	113.6 ± 39.2	108.9 ± 33.0	**<.001**
hs‐CRP, mg	0.11 [0.05‐0.33]	0.20 [0.07‐0.67]	0.11 [0.05‐0.30]	0.08 [0.03‐0.20]	**<.001**
eGFR, mL/min/1.73 m^2^	68.8 ± 23.8	65.9 ± 24.6	68.9 ± 23.4	71.8 ± 22.9	**<.001**

*Note*: Values significantly different among groups were indicated in bold.

Abbreviations: ACEI/ARB, angiotensin‐converting enzyme inhibitor/angiotensin receptor blocker; ACS, acute coronary syndrome; ApoA1, apolipoprotein A1; ApoB100, apolipoprotein B100; ApoE, apolipoprotein E; BMI, body mass index; CCB, calcium channel blocker; eGFR, estimated glomerular filtration rate; HbA1c, glycated hemoglobin; HDL‐C, high density lipoprotein‐cholesterol; hs‐CRP, high‐sensitivity C‐reactive protein; LAD, left anterior descending coronary artery; LCX, left circumflex coronary artery; LDL‐C, low density lipoprotein‐cholesterol; LMT, left main trunk coronary artery; Non‐FBG, nonfasting blood glucose; RCA, right coronary artery; SVG, saphenous vein graft; TC, total cholesterol; TG, triglycerides.

### 3.2 Higher incidences of all‐cause and cancer mortalities following PCI in patients with lower ApoA1

During the follow‐up period that was up to 10 years since the PCI, 531 all‐cause deaths occurred, and noncardiovascular death (non‐CV death) was predominant (n = 337, 63.5% out of all deaths). The numbers of identified total, GI and lung cancer mortalities after PCI were 174 (32.8%), 86 (16.2%) and 40 (7.5%), respectively. Moreover, the proportion of cancer deaths had increased relatively over time, when participants were equally divided into three cohorts by date of PCI procedure: first period (January 7, 2000 to March 13, 2006): 31.3%, second period (March 14, 2006 to September 8, 2011): 34.0% and third period (September 9, 2011 to February 3, 2018): 35.2%, respectively.

**FIGURE 1:**
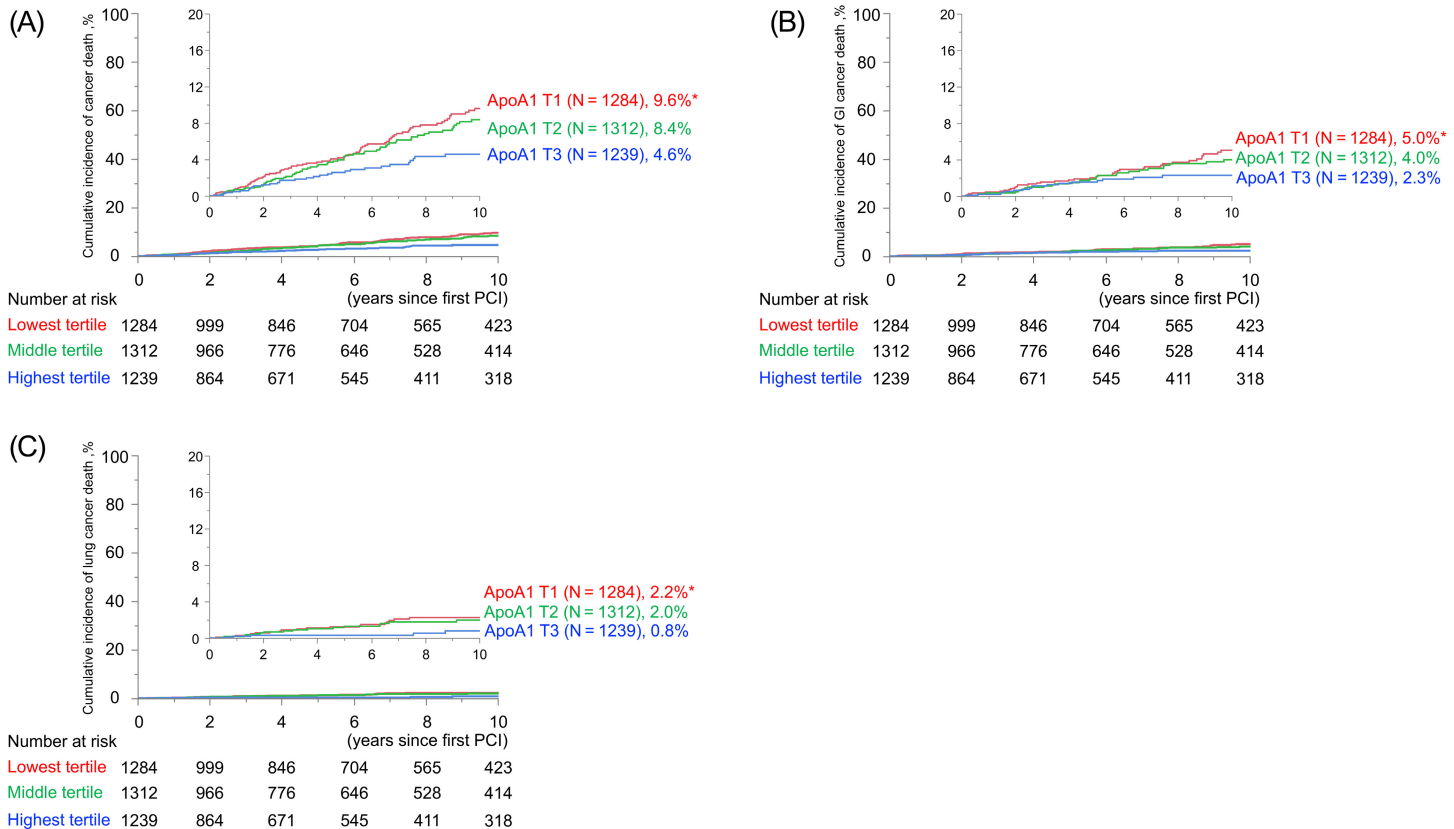
Cumulative cancer mortality rates in groups divided by tertiles of preprocedural ApoA1. Cumulative rates of (A) total, (B) GI, and (C) lung cancer mortality. ApoA1, apolipoprotein A1; GI, gastrointestinal [Color figure can be viewed at wileyonlinelibrary.com]

The crude incidences of total, GI, and lung cancer mortalities per 1000 person‐years were significantly higher in patients with lower ApoA1 across the groups (Table 2). Moreover, unadjusted Kaplan‐Meier analyses with log‐rank comparisons showed significantly higher rates of cumulative incidences of total, as well as both GI and lung cancer mortalities in the lowest tertile of the ApoA1 group compared to those in the highest tertile (Figure 1A‐C). In contrast, no significant differences in all types of cancer mortalities were observed in the groups divided by the tertiles of HDL‐C (38, 48 mg/dL, respectively), despite a strong correlation between serum levels of ApoA1 and HDL (Figure S2A‐C).

**TABLE 2:** Overall incidence of adverse events (per 1000 person‐years)

	Overall (n = 3835)	Lowest tertile (T1) (ApoA1 ≤ 110) (n = 1284)	Middle tertile (T2) (110 < ApoA1 ≤ 130) (n = 1312)	Highest tertile (T3) (ApoA1 > 130) (n = 1239)	*P*‐value
All‐cause death, n (/1000 person‐years)	531 (24.4)	231 (29.4)	173 (23.1)	127 (19.7)	**<.001**
Noncardiovascular death, n (/1000 person‐years)	337 (15.5)	149 (18.9)	121 (16.2)	67 (10.4)	**<.001**
Cancer death, n (/1000 person‐years)	174 (8.0)	78 (9.9)	64 (8.6)	32 (5.0)	**<.001**
Gastrointestinal cancer death, n (/1000 person‐years)	86 (3.9)	39 (5.0)	30 (4.0)	17 (2.6)	**.016**
Lung cancer death, n (/1000 person‐years)	40 (1.8)	19 (2.4)	16 (2.1)	5 (0.8)	**.022**
Cardiovascular death, n (/1000 person‐years)	194 (8.9)	82 (10.4)	52 (6.9)	60 (9.3)	**.017**

*Note*: Values significantly different among groups were indicated in bold.

### 3.3 Adjusted prognostic implications of serum ApoA1 level for cancer mortalities

**FIGURE 2:**
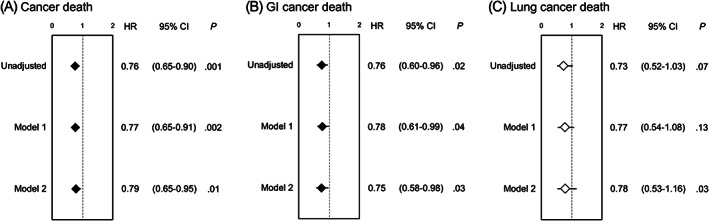
Unadjusted and multivariate adjusted (Models 1 and 2) prognostic impact of preprocedural ApoA1 level in patients for total, GI, and lung cancer mortalities. Hazard ratios (HR) and 95% confidence intervals (95% CIs) for (A) total, (B) GI, and (C) lung cancer mortalities, respectively. For multivariate analysis, Model 1 was adjusted by age, sex, and any smoking history, and Model 2 additionally included hypertension, diabetes, serum LDL‐C level, chronic kidney disease, BMI, use of ACEI/ARB, use of statins, and serum hs‐CRP level, respectively. Closed rhombuses indicate a significant (*P* < .05) association of 1 − SD elevation of ApoA1 with endpoints. ACEI/ARB, angiotensin converting enzyme inhibitors/angiotensin receptor blockers; ApoA1, apolipoprotein A1; BMI, body mass index; GI, gastrointestinal; hs‐CRP, high‐sensitivity C‐reactive protein; LDL‐C, low density lipoprotein cholesterol

**FIGURE 3:**
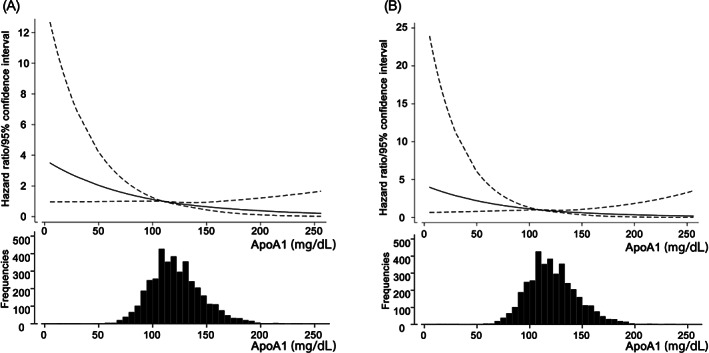
Cubic spline curves and histograms of serum ApoA1 level for the risk of total and GI cancer mortalities following PCI. Hazard ratios (solid lines) and 95% confidence intervals (dotted lines) for total (A) and GI (B) cancer mortalities as a reference ApoA1 value of 110 mg/dL and histograms of serum level of ApoA1. The Cox model included age, sex, any smoking history, hypertension, diabetes, serum LDL‐C level, chronic kidney disease, body mass index, use of ACEI/ARB, use of statins and serum hs‐CRP level (Model 2). ACEI/ARB, angiotensin converting enzyme inhibitors/angiotensin receptor blockers; ApoA1, apolipoprotein A1; GI, gastrointestinal; hs‐CRP, high‐sensitivity C‐reactive protein; LDL‐C, low density lipoprotein cholesterol; PCI, percutaneous coronary intervention

To further address the impact of serum ApoA1 level on cancer mortality in patients after PCI, univariate‐unadjusted and multivariate‐adjusted Cox proportional hazard analyses calculated the hazard ratios (HRs) of a 1 − SD higher ApoA1 level as a continuous valuable, for predicting total, GI, and lung cancer mortalities. Univariate analyses showed that a 1 − SD higher ApoA1 level at PCI procedure was significantly associated with reduced risk of total and GI cancer mortalities, while not significantly associated with lung cancer mortality. Similarly, multivariate analyses adjusted by age, gender, and any smoking history (Model 1) showed a significant risk reduction by a 1 − SD higher ApoA1 level for total and GI cancer mortalities, while no significant association with lung cancer mortality. Moreover, a significant relative risk reduction by higher ApoA1 was maintained in a Cox model that further included BMI and serum hs‐CRP level and taking ACEIs/ARBs and statins at PCI in addition to classical risk factors for atherosclerotic cardiovascular disease (Model 2) (Figure 2). Moreover, as illustrated by the cubic spline curve of serum ApoA1 level, the risk for future cancer mortality following PCI appeared to be inversely associated and the relationship was linear. The inverse linear correlation in the risk of GI cancer mortality with serum ApoA1 level was preserved (Figure 3A,B).

## 4 DISCUSSION

The present study involving 3835 patients who underwent PCI demonstrated that the incidence of cancer mortality, GI cancer mortality in particular, was significantly higher in patients with lower ApoA1 at PCI procedure. In contrast, there were no significant differences among the Kaplan‐Meier curves of any cancer mortalities when the participants were divided by HDL tertiles. Multivariate Cox proportional hazard analyses adjusted by traditional risk factors, as well as those previously indicated to associate with cancer incidence and mortality, showed lower ApoA1 increased the risk of total and GI cancer mortalities. Moreover, cubic spline curves fitted in the Cox model illustrated a linear decrease in the risk of total and GI cancer mortalities in accordance with elevating ApoA1.

PCI has matured and both procedural and long‐term clinical outcomes have substantially improved since its introduction, due to progress in the technology of devices, procedural techniques, and in particular, evidence‐based effective pharmacological strategies in the secondary prevention of CAD, such as statins and antiplatelet medications.^29^, ^30^ In contrast, noncardiovascular death in patients after PCI is increasing,^8^ although cardiovascular death has been a primary outcome measure in the vast majority of randomized trials, as well as cohort analyses of patients who underwent PCI. A registry‐based observational study showed noncardiovascular death occurred in approximately 14% of patients in the 6 years following PCI, while cardiovascular death occurred in 8%.^31^ Similarly, a metaanalysis from 21 randomized trials of PCI patients between 1999 and 2016 demonstrated that approximately half of all‐cause death was not cardiovascular‐related.^32^ The rise in noncardiovascular mortality was driven by the increased comorbidities, which have been mainly attributable to cancer.^8^ From the opposite perspective, patients with cancer have constituted a significant proportion in the PCI population who have an increased risk for cardiovascular morbidity and mortality.^33^ In our study, the proportion of noncardiovascular death to all‐cause death in 10 years after PCI was more than two‐thirds in the later periods (2000‐2006: 58.1%, 2006‐2011: 68.9%, 2011‐2018: 68.5%). Similarly, cancer death increased slightly across periods from 31.3% up to 35.2%. These findings have implications for treating patients with CAD undergoing PCI in terms of considering the comprehensive management of noncardiovascular comorbidities, and clinicians should be particularly cautious with respect to whether the patient is complicated by malignant diseases, which offers significant benefits in combination with attention to cardiovascular disease.^32^ Compelling evidence indicates that HDL has protective roles against atherosclerosis.^14^, ^34^ Therefore, a reduced level of serum HDL‐C has been considered as an independent negative prognostic factor in cardiovascular outcome for more than four decades.^15^, ^35^ In addition to the pivotal roles of HDL in the process of cholesterol efflux from lipid laden cells, which associates with the preventative effects of atherosclerosis,^36^ experimental evidence has suggested that HDL regulates innate and adaptive immune responses and has in turn antioxidative and antiinflammatory properties which are partly independent from the effects on cholesterol kinetics.^37^ In particular, ApoA1, a principal constituent protein of HDL,^38^ is a major enzyme involved in the antioxidant and antiinflammatory properties of HDL.^39^ As inflammation and oxidative stress play central roles not only in atherosclerosis, but also in the carcinogenesis of various cancers,^40^, ^41^ the association between decreased HDL or ApoA1 and increased risk of cancer has been indicated through the dysfunction of such beneficial properties.^42^ In light of the pathogenesis of cancer development, the HDL‐ApoA1 axis has been postulated to potentially modulate proliferative and inflammatory pathways via its immunomodulatory, antioxidative, antiapoptotic, and antiinflammatory properties.^43^, ^44^ Indeed, a number of clinical studies have discovered associations between the reduced levels of serum ApoA1 and increased risk of many types of cancer in general population.^45^ Moreover, previous observational studies indicated that patients with cancer or cancer history had increased risk of in‐hospital mortality following PCI^33^ and that elevated ratio of white blood cell count to ApoA1 at PCI was associated with all‐cause and cardiac mortalities.^46^ However, the long‐term impact of ApoA1/HDL axis regarding cancer mortality in PCI patients has not been previously evaluated. Moreover, experimental studies have suggested that ApoA1, rather than total HDL, has anticancer properties which potentially inhibit proliferation or growth of cancer cells,^47^ including a study demonstrating reduced transcriptional levels of ApoA1 in hepatocellular carcinoma (HCC) compared to normal liver tissue.^48^ The present study complements and extends the results from these prior clinical and experimental studies. However, in contrast, Mendelian randomization analyses have failed to show a causal association of HDL cholesterol and risk of breast and colorectal cancer.^49^, ^50^ Therefore, the association between the HDL‐ApoA1 axis and cancer risk remains under debate and is yet to be fully evaluated. Moreover, it is also unclear whether the risk of cancer is predominantly associated with HDL or Apo A1. In the present study involving patients with established atherosclerotic coronary artery disease, the effect of preprocedural ApoA1 level on total cancer mortality was significant compared to HDL‐C level.

With respect to the association of HDL and ApoA1 with the risk of a specific cancer type, analyses from the Women's Health Study identified an inverse association with colorectal and lung cancer,^20^ while a large‐scale population‐based study involving more than 10 000 individuals demonstrated that the associations were relatively less in GI and respiratory cancers compared to hematological and neurologic cancers.^21^ In our study, the association of ApoA1 with a specific type of cancer mortality was more significantly enhanced in GI cancer mortality than in lung cancer mortality. As the number of mortalities due to malignancies was limited in the present study, the prognostic associations of death by hematological and neurologic cancers could not be assessed. In light of mechanistic insights into the association between reduced ApoA1 and the increased risk of cancer mortality, a significant inverse correlation between the extent of chronic systemic inflammation represented by hs‐CRP level and ApoA1 might be a possible explanation in our study.

## 5 LIMITATIONS

The present study has several limitations. First, the retrospective nature of the study may not be suitable to infer causality, although data was prospectively collected. Moreover, even though the findings in our study were adjusted by multivariate models, residual confounding factors which might explain the causation cannot be excluded. Second, the single‐center setting involving only Japanese patients may limit the generalizability of the present findings, and therefore, we may need to be careful when applying the present findings to different populations in different situations. Third, although data regarding mortality was precisely and rigorously corrected via electrical medical records and by periodic phone calls, data on cancer incidence is not available, indicating that the impacts of HDL and ApoA1 levels on cancer incidence were not evaluated in our study. Finally, because of the relatively small number of total participants and endpoint incidence, evaluating the contribution of ApoA1 to the risk of specific cancer types was underpowered. Therefore, further investigations with larger sample sizes are needed to assess the associations of ApoA1 with specific types of cancer.

Despite these limitations, our study demonstrated that reduced preprocedural ApoA1 was independently associated with the future risk of total and GI cancer mortality following PCI. Because cancer has recently become dominant as a cause of mortality in patients following PCI, serum ApoA1 level might be a useful prognostic tool to predict outcomes in patients who are going to undergo PCI.

## AUTHOR CONTRIBUTIONS

Hiroki Nishiyama, Takehiro Funamizu and Hiroshi Iwata contributed to the research idea, data analysis/interpretation, study design, statistical analysis and preparation of the manuscript. Hiroki Nishiyama, Takehiro Funamizu, Yuichi Chikata, Shinichiro Doi, Hirohisa Endo, Hideki Wada, Ryo Naito, Manabu Ogita, Yoshiteru Kato, Iwao Okai, Tomotaka Dohi, Takatoshi Kasai and Kikuo Isoda contributed to data acquisition. Hiroshi Iwata, Shinya Okazaki, Katsumi Miyauchi and Tohru Minamino contributed to supervision. Tohru Minamino approved the final version of the manuscript for submission. The work reported in the paper has been performed by the authors, unless clearly specified in the text.

## CONFLICT OF INTEREST

The authors declare that they have no conflicts of interest.

## ETHICS STATEMENT

Our study was performed in accordance with the Declaration of Helsinki and approved by the Institutional Review Board (IRB) of Juntendo University (IRB number: 17‐170). A prospective registry database of patients who underwent any PCI at Juntendo University Hospital, Tokyo, Japan (Juntendo Physicians' Alliance for Clinical Trial: J‐PACT) is publicly registered (University Medical Information Network Japan—Clinical Trials Registry, UMIN‐CTR 000035587). Written informed consent was obtained from all participants for the J‐PACT registry.

## Supporting information


**Appendix S1** Supporting Information

## Data Availability

All data used in our study will be available upon request.
