# Supplementary material for: Low apolipoprotein A1 was associated with increased risk of cancer mortality in patients following percutaneous coronary intervention: A 10‐year follow‐up study
Source: Int J Cancer. 2022 Jul 7;151(9):1482–90. doi: 10.1002/ijc.34164 (PMC9540779; doi:10.1002/ijc.34164)
Supplement: Supplementary file 1 — Appendix S1 Supporting Information [file IJC-151-1482-s001.pdf]

Title

Low Apolipoprotein A1 was associated with increased risk of cancer mortality in patients following percutaneous coronary intervention: A 10-year follow-up study

Authors

Hiroki Nishiyama, M.D., Takehiro Funamizu, M.D., Ph.D, Hiroshi Iwata, M.D., Ph.D, Hirohisa Endo, M.D., Ph.D, Yuichi Chikata, M.D., Ph.D, Shinichiro Doi, M.D., Ph.D, Hideki Wada, M.D., Ph.D, Ryo Naito, M.D., Ph.D, Manabu Ogita, M.D., Ph.D, Yoshiteru Kato, M.D., Ph.D, Iwao Okai, M.D., Ph.D, Tomotaka Dohi, M.D., Ph.D, Takatoshi Kasai, M.D., Ph.D, Kikuo Isoda, M.D., Ph.D, Shinya Okazaki, M.D., Ph.D, Katsumi Miyauchi, M.D., Ph.D, Tohru Minamino, M.D., Ph.D

Table of contents

Supplemental Figure 1: Consort diagram of the present study……………………………………..2
Supplemental Figure 2: Cumulative cancer mortality rates in groups divided by tertiles of preprocedural HDL-C……………………………………………………………………………3

Supplemental Figure 1: Consort diagram of the present study

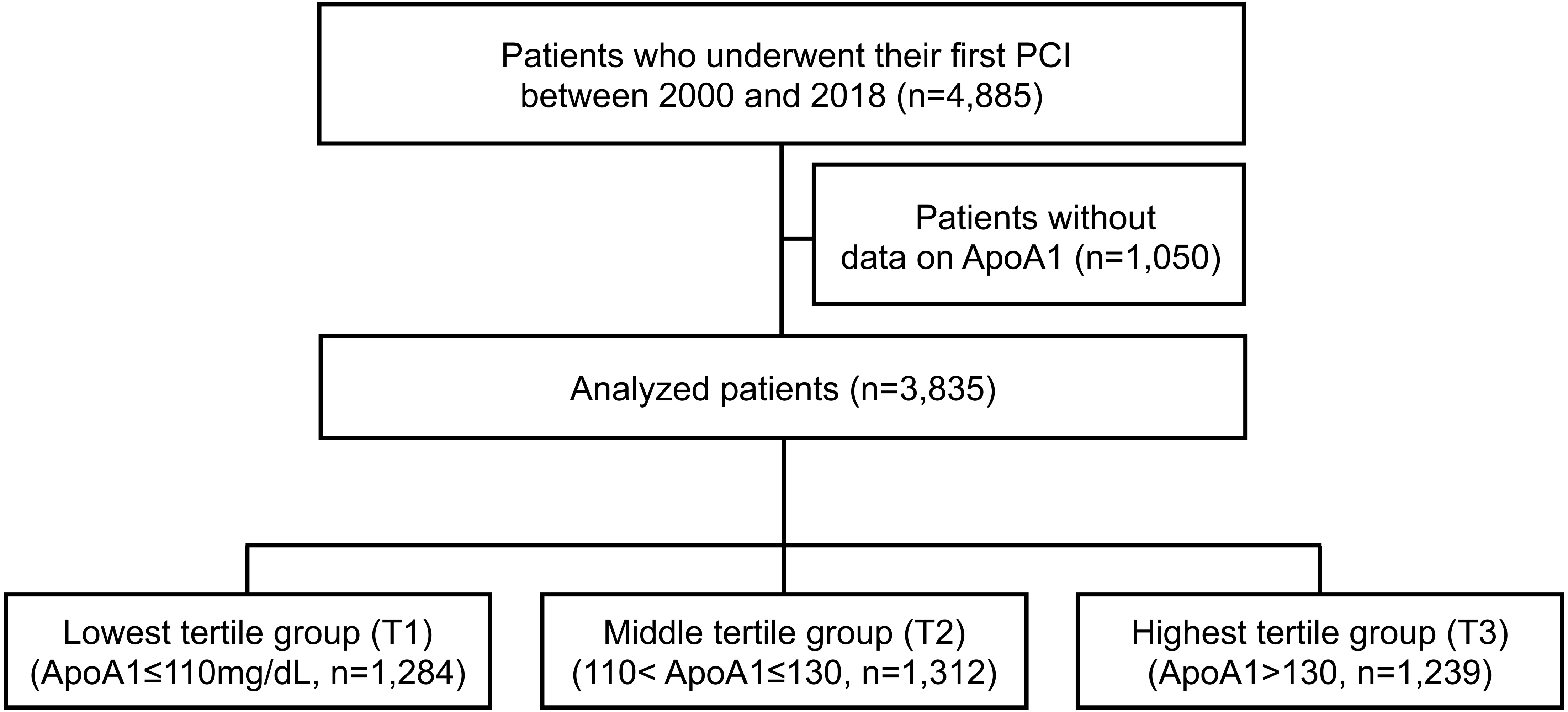

Patients who underwent their first PCI at Juntendo University Hospital between 2000 and 2018 were enrolled in the present study. Patients without data on ApoA1 were excluded.
Abbreviations: ApoA1 = Apolipoprotein A1; PCI = percutaneous coronary intervention.

Supplemental Figure 2: Cumulative cancer mortality rates in groups divided by tertiles of preprocedural HDL-C

(A)

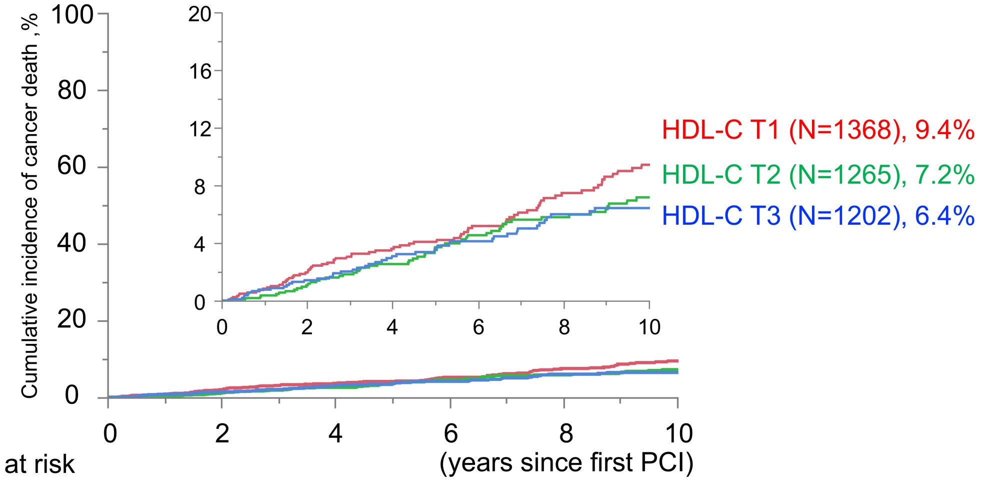

Number at risk						
Lowest tertile	1368	1019	815	662	522	400
Middle tertile	1265	946	780	650	528	400
Highest tertile	1202	864	698	583	454	355

(B)

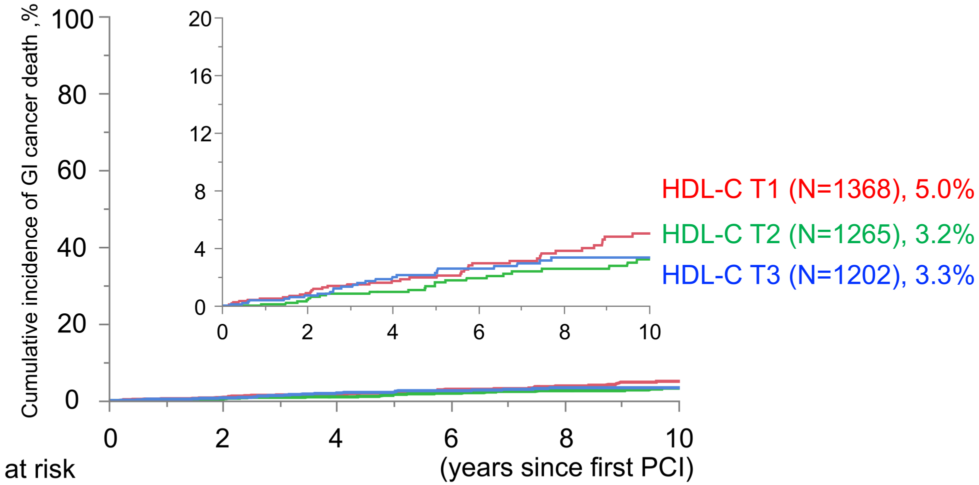

Number at risk						
Lowest tertile	1368	1019	815	662	522	400
Middle tertile	1265	946	780	650	528	400
Highest tertile	1202	864	698	583	454	355

(C)

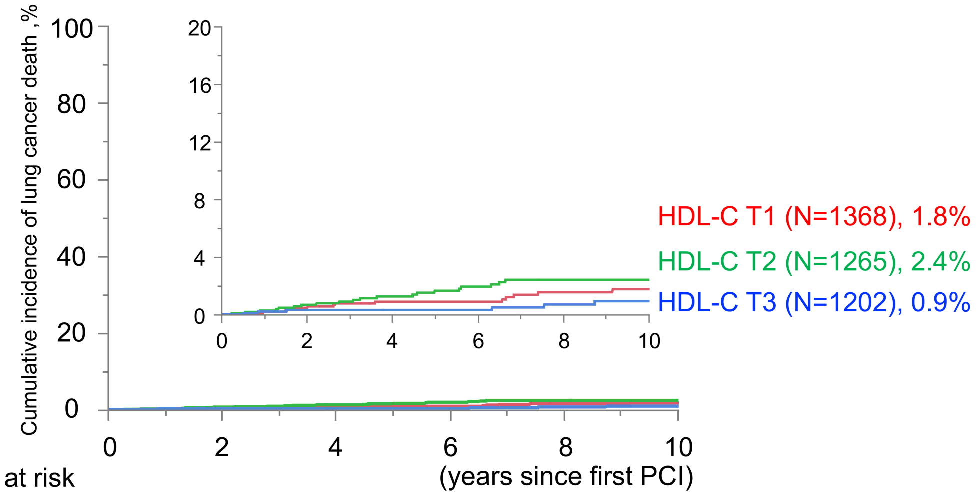

Number at risk						
Lowest tertile	1368	1019	815	662	522	400
Middle tertile	1265	946	780	650	528	400
Highest tertile	1202	864	698	583	454	355

Cumulative rates of A total, B GI, and C lung cancer mortality.
Abbreviations: HDL-C = High-density lipoprotein cholesterol; GI = gastrointestinal.
